# Enhanced Non-Associative Long-Term Potentiation in Immature Granule Cells in the Dentate Gyrus of Adult Rats

**DOI:** 10.3389/fnsyn.2022.889947

**Published:** 2022-05-30

**Authors:** Natalia A. Simonova, Maxim A. Volgushev, Alexey Y. Malyshev

**Affiliations:** ^1^Institute of Higher Nervous Activity and Neurophysiology of Russian Academy of Sciences, Moscow, Russia; ^2^Department of Psychological Sciences, University of Connecticut, Storrs, CT, United States

**Keywords:** adult neurogenesis, dentate gyrus, granule cells, GABA, non-associative potentiation, heterosynaptic plasticity

## Abstract

The dentate gyrus is one of the few sites of neurogenesis in the adult brain. Integration of new-generated granule cells into the hippocampal circuitry provides a substrate for structural plasticity, fundamental for normal function of adult hippocampus. However, mechanisms of synaptic plasticity that mediate integration of new-generated granule cells into the existing circuitry remain poorly understood. Especially mechanisms of plasticity at GABA-ergic synapses remain elusive. Here, we show that postsynaptic spiking without presynaptic activation can induce heterosynaptic, non-associative plasticity at GABA-ergic inputs to both immature and mature granule cells. In both immature and mature neurons, plastic changes were bidirectional and individual inputs could express long-term potentiation (LTP) or long-term depression (LTD), or do not change. However, properties of non-associative plasticity dramatically change with maturation of newly generated granule cells: while in immature cells there was a clear predominance of non-associative LTP and net potentiation across the inputs, in mature neurons, potentiation and depression were balanced with no net change on average. We conclude that GABA-ergic inputs to granule cells are plastic, and that the rules for induction of non-associative plasticity change with maturation. We propose that potentiation-biased non-associative plasticity of GABA-ergic transmission might help to counter-balance an increase of excitatory drive that is facilitated by enhanced LTP at glutamatergic synapses in maturating granule cells. Such mechanism might help to build a strong GABA-ergic input to surviving active new cells, necessary for normal function of mature granule cells, which operate under a tight inhibitory control and generate sparse spiking activity.

## Introduction

The dentate gyrus of the hippocampal formation is one of the few brain regions in which new neurons are generated throughout the life (Altman and Das, 1965; Eriksson et al., 1998; Kempermann et al., 2000). Generation of new granule cells and their integration in the existing circuitry is a central element of structural plasticity in the adult hippocampus, fundamental for its normal function. Newly generated granule cells of the dentate gyrus play an important role in learning and formation, preservation, and interference of memories (Deng et al., 2010; Arruda-Carvalho et al., 2011; Burghardt et al., 2012; Miller and Sahay, 2019; Trinchero et al., 2019a,b), including fear conditioning (Pan et al., 2012) and contextual discrimination (Zhuo et al., 2016). After generation, new granule cells undergo several stages during which they get integrated in the existing circuitry. At each stage, maturating cells are characterized by specific morphological and electrophysiological properties and patterns of synaptic inputs (Wang et al., 2000; Schmidt-Hieber et al., 2004; Markwardt et al., 2009; Mongiat et al., 2009; Marin-Burgin et al., 2012; Trinchero et al., 2019a). Notably, at early stages of maturation, GABA-ergic inputs to granule cells are depolarizing and play a pivotal role in activation and survival of newly generated neurons (Ge et al., 2007a; Mongiat and Schinder, 2011; Dieni et al., 2013). After ~8 weeks, new cells become mature, fully integrated in the neuronal circuitry, with morphological, membrane, and synaptic properties indistinguishable from those of other granule cells.

Despite recent insights into the role and features of newly generated granule cells, mechanisms of synaptic plasticity that mediate the establishment and modification of excitatory and inhibitory inputs to adult-born new granule cells during their maturation and integration into existing pattern of connectivity remain poorly understood. Prior research revealed an enhanced long-term potentiation (LTP) at excitatory glutamatergic synapses to immature granule cells during the first few weeks of maturation (Snyder et al., 2001; Schmidt-Hieber et al., 2004; Ge et al., 2007b). However, plasticity at inhibitory GABA-ergic synapses remains unexplored. This represents a major gap in our knowledge, especially taking into account that in new granule cells, responses to GABA appear before responses to glutamate, are depolarizing during the first 2–3 weeks, and might be vital for activation of new neurons, which is necessary for their survival (Ge et al., 2006, 2007b; Dieni et al., 2013). Moreover, because maintenance of excitatory/inhibitory balance is crucial for normal neuronal operation (Wehr and Zador, 2003; Klausberger and Somogyi, 2008; Ozeki et al., 2009; Dorrn et al., 2010), and tight inhibitory control is a necessary prerequisite for sparse spiking of mature granule cells and sparse encoding in the dental gyrus (Burghardt et al., 2012; Dieni et al., 2013; McAvoy et al., 2016), development of excitatory inputs has to be accompanied by a build-up and strengthening of inhibition. Mechanisms that may strengthen GABA-ergic synapses to keep up with an increase of excitatory drive facilitated by enhanced LTP at excitatory synapses remain elusive. Evidence from neocortical pyramidal neurons shows that plastic changes at GABA-ergic synapses could be induced by bursts of postsynaptic spikes, without the need for presynaptic activation (Kurotani et al., 2003, 2008; Inagaki et al., 2008; Kuczewski et al., 2008; Lourenço et al., 2014). Here, we asked if plastic changes could be induced by postsynaptic activity at GABA-ergic inputs to new-born granule cells, and whether the rules for induction of such non-associative plasticity change with maturation of granule cells.

## Methods

All experimental procedures were in compliance with the Guide for the Care and Use of Laboratory Animals published by the National Institute of Health and were approved by the Ethical Committee of the Institute of Higher Nervous Activity and Neurophysiology, Russian Academy of Sciences.

### Preparation of Acute Brain Slices

Acute brain slices were prepared using conventional techniques (Ting et al., 2014). Briefly, 1.5 to 2-month-old (250–300 g) Long-Evans rats of both sexes were deeply anesthetized with isoflurane and decapitated. The hippocampus was rapidly removed and immersed in ice-cold oxygenated sucrose-based solution, in mM: 83 NaCl, 25 NaHCO_3_, 2.7 KCl, 1 NaH_2_PO_4_, 0.5 CaCl_2_, 3.3 MgCl_2_, 20 glucose, 71 sucrose, bubbled with 95% O_2_/5% CO_2_. The oxygenated sucrose-based solution was used during preparation of slices in the slice incubator. Transverse slices of the hippocampus (350 μm) were cut using a vibratome (VT1200S, Leica, Germany) and incubated for 45–60 min at 34°C for recovery. After that, slice incubation chamber was moved to room temperature. For recording, individual slices were transferred into a recording chamber mounted on an Olympus BX-50WI microscope equipped with IR-DIC optics. ACSF solution for recording contained, in mM, 125 NaCl, 25 NaHCO_3_, 25 glucose, 3 KCl, 1.25 NaH_2_PO_4_, 2 CaCl_2_, 1 MgCl_2_, bubbled with 95% O_2_/5% CO_2_, pH 7.4. Recordings were made at 30–32°C.

### Electrophysiology

Whole-cell recordings with patch electrodes were obtained from the granule neurons in the inner portion of granule cell layer of the dentate gyrus using DIC-optics and infrared videomicroscopy. Immature cells were visually pre-selected by the smaller size and slightly curved shape of their somata, and visible processes oriented not exactly parallel to the processes of the majority of nearby cells. Such cells were often located at the border or slightly out of the cell layer. Visual pre-selection increased the yield of immature cells, but the final classification was made based on an analysis of electrophysiological properties, morphology, and immunostaining as described below. The patch electrodes were filled with a potassium gluconate based solution (130 mM potassium gluconate, 20 mM KCl, 4 mM Mg-ATP, 0.3 mM Na_2_-GTP, 10 mM sodium phosphocreatine, 10 mM Hepes) and had a resistance of 4–6 MΩ. Recordings were made with a MultiClamp 700B amplifier (Molecular Devices, USA) in the current clamp or voltage clamp mode. After amplification and low-pass filtering at 10 kHz, the data were digitized at 10 kHz and fed into a computer using Digidata 1500 interface and pCLAMP software (both from Molecular Devices, USA). Synaptic responses were evoked using monopolar glass stimulation electrode, filled with ACSF, and placed in the outer portion of the granule layer, close to the border to the inner molecular layer of the dentate gyrus. Stimulation strength was adjusted to evoke PSP responses with amplitudes of 2–6 mV. In some experiments, as indicated, blockers of GABA_A_ or AMPA receptors were added to the perfusion medium. GABA_A_-receptor blocker bicuculline methiodide (Sigma-Aldrich, USA) was used in the final concentration of 10 μM, and AMPA-receptor blocker CNQX (water-soluble form, disodium salt; Sigma-Aldrich, USA) was used in the final concentration of 10 μM.

### Immunohistochemistry and Intracellular Staining

A subset of recorded neurons was labeled for morphological identification and immunochemical staining. For labeling, Neurobiotin (Vector Laboratories, USA) was added to the intracellular solution at a concentration of 0.2%. Cells were patched and held for 30–60 min to let the dye diffuse into the cell; then, the recording pipette was withdrawn and the cells were allowed to survive for 5–20 min. After that, slices were immediately transferred in a fixative containing 4% paraformaldehyde in PBS and fixed overnight at +4°C. Next, after washing 3 times in PTA (0.5% Triton X-100 and 0.01% sodium azide in PBS), free floating slices were incubated for 48–72 h in Streptavidin-TexasRed (1:100, Vector Laboratories, Burlingame CA, USA) followed by 3 times rinse in PTA. For immunochemical staining, sections with labeled neurons were incubated with blocking solution (10% normal goat serum, 0.5% Triton X-100, and 0.01% sodium azide in PBS) for 2 h followed by incubation with anti-polysialic acid-NCAM (PSA-NCAM) monoclonal mouse antibodies (1:100, Millipore, USA) in blocking solution for 48–72 h at +4°C. After 5 rinses in PTA, sections were treated with Alexa-488 conjugated goat anti-mouse polyclonal antibodies (1:50, Invitrogen, USA) for 48–72 h at +4°C. Then, the sections were rinsed in PTA, stained with DAPI, and glass-mounted using AquaPolymount (Polysciences, USA). Specimens were viewed and images were acquired using a Zeiss Axioscope2 epifluorescent microscope or a laser-scanning Cerna-based confocal microscope (ThorLabs, USA).

### Data Processing and Statistical Analysis

Electrophysiological data were processed using CLAMPEX (Molecular Devices, USA) software, Excel (MS Office 2016), and custom-written scripts in MATLAB (The MathWorks, Natick MA, USA) and R (version 3.4.0, 2017-04-21, The R Foundation for Statistical Computing). Input resistance (R_in_) and membrane time constant were measured using responses to small amplitude pulses of hyperpolarizing current (-50 pA, 100 ms). Amplitude of PSP responses was measured as the difference between the mean membrane potential in two time windows, one placed immediately before the PSP onset, and another one on the PSP rising slope just before the peak. Measuring windows (0.5–2 ms width, 1.3–5 ms separation) were set for each cell according to PSP kinetics; windows for the second PSP in paired-pulse paradigm had same width and separation as those for the first PSP, but shifted by the interpulse interval (50 ms). Paired-pulse ratio (PPR) was calculated as the ratio of the amplitude of response evoked by the second pulse to the amplitude of response evoked by the first pulse. Inverse of the coefficient of variation (CV^−2^) was calculated using responses to the first pulse in the paired-pulse paradigm. For calculating the significance of response amplitude changes at individual inputs, we used a *t*-test (MATLAB) to compare the amplitude of responses in control and after application (or omission, see below) of plasticity induction protocol. Cells with significant response changes (*p* < 0.05) were considered expressing LTP or LTD; cells with no significant response changes were considered as expressing no plasticity. For comparisons of groups of cells (with each cell contributing one data point to a group), we used a *t*-test for a comparison of the means, non-parametric Kolmogorov-Smirnov test for a comparison of the distributions, *F*-test for a comparison of the variances (see **Figures 4**, **5** and related text for detail), and a chi-square test for a comparison of the frequency of occurrence of LTP and LTD.

**Figure 1:**
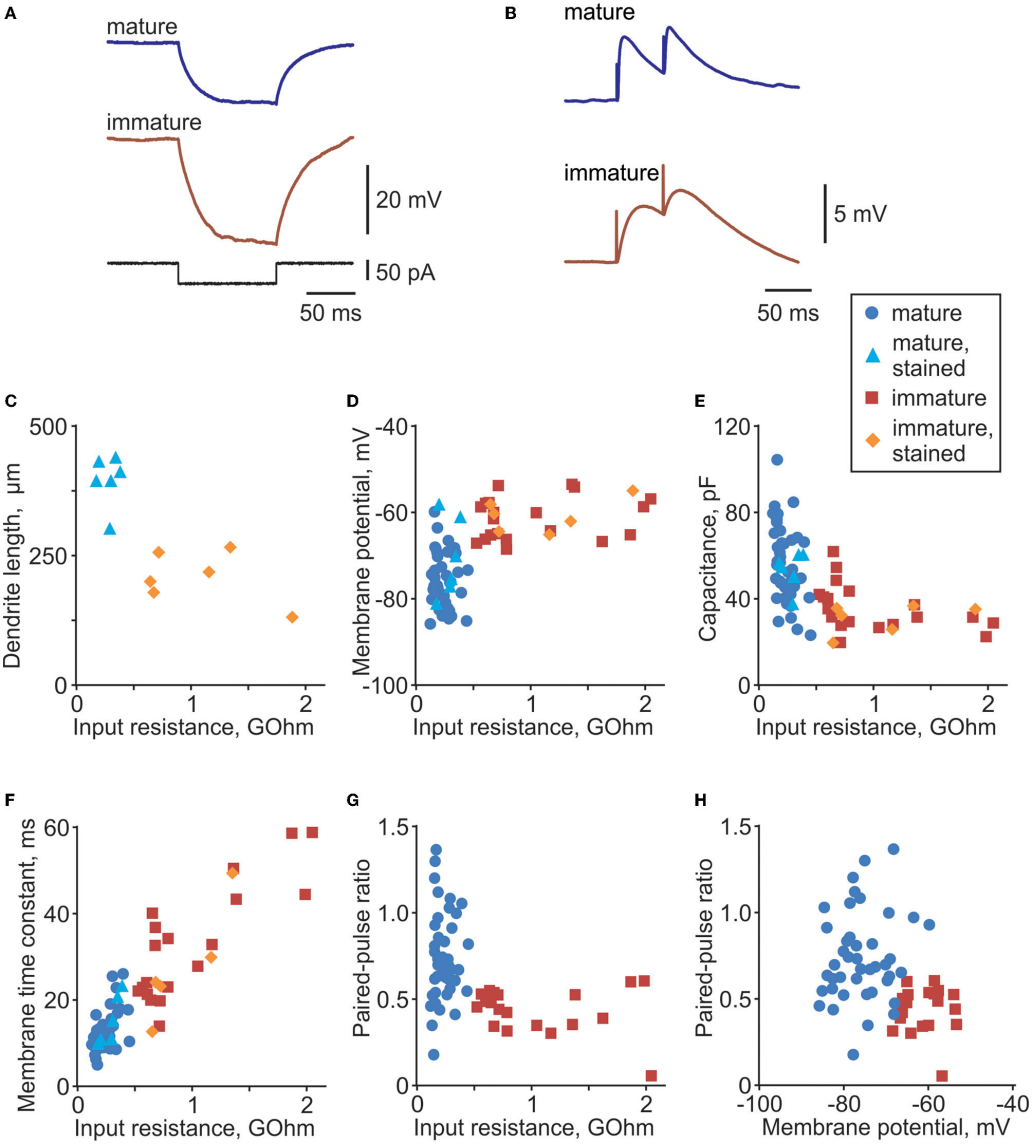
Distinct electrophysiological properties of mature and immature cells in rat dentate gyrus. **(A)** Membrane potential response to injection of a −50 pA current step in example mature and immature neurons in dentate gyrus. Responses to negative current steps were used to measure input resistance and membrane time constant. **(B)** Example PSP responses in a mature and an immature neuron evoked by paired-pulse stimulation. **(C)** Dendrite length (distance between the most distant dendrite and the soma) plotted against input resistance for 6 mature (PSA-NCAM immunonegative, light blue triangle symbols) and 6 immature (PSA-NCAM immunopositive, orange diamond symbols) cells. **(D–F)** Membrane potential **(D)**, cell capacitance **(E)**, and membrane time constant **(F)** plotted against input resistance for morphologically identified mature (*n* = 6, light blue triangle symbols) and immature cells (*n* = 6, orange diamond symbols), and electrophysiologically classified mature (*n* = 51, blue circle symbols) and immature (*n* = 20, dark red square symbols) cells. **(G,H)** Paired-pulse ratio plotted against input resistance **(G)** and membrane potential **(H)** for electrophysiologically classified mature (*n* = 51, blue circle symbols) and immature (*n* = 20, dark red square symbols) cells.

Classification of cells as mature or immature was made using quadratic discriminant analysis (scripts in R, functions qda.fit and qda.pred from the library MASS), and verified by expert evaluation. Classification algorithm was trained using electrophysiological data from morphologically and immunochemically identified immature and mature cells, and then the trained algorithm was applied to classify all recorded cells. The assignment of cells into the immature or mature groups was verified by expert evaluation using scatterplots of pairs of electrophysiological parameters (see Figure 1 and Results for detail). Only unambiguously and reliably classified cells were used for the final analysis.

Thus, all cells included in the analysis fulfilled the following criteria: (1) stability of the membrane potential and input resistance throughout the recording (changes <20% of control), (2) stability of PSP amplitudes during the control period, (3) stability of the onset latency and kinetics of the rising slope of the PSP, and (4) reliable classification as immature or mature.

Throughout the text, mean values are given together with SD; differences are considered significant at *p* < 0.05; *p*-values >0.001 are given in full, or as >0.1; *p*-values < 0.001 are given as *p* < 0.001.

### Data Availability

Original data and processing codes are available from the corresponding author upon request.

## Results

### Distinct Electrophysiological Properties of Mature and Immature Granule Cells in the Dentate Gyrus

We made whole-cell recordings from *n* = 86 granule cells in the inner portion of the granule cell layer of the dentate gyrus. This area contains immature cells that are newly generated from neural stem cells located in the adjacent subgranular zone (Gage, 2000; van Praag et al., 2002). Immature cells have a rudimentary dendritic tree, smaller somata, and receive fewer synaptic contacts than mature neurons. A characteristic feature of immature cells is high input resistance (R_in_) (Schmidt-Hieber et al., 2004; Mongiat et al., 2009; Trinchero et al., 2019a); therefore, we used input resistance measured from responses to small amplitude steps of hyperpolarizing current (Figure 1A) as a criterion for initial preliminary discrimination between immature and mature cells during the experiment. Classification of immature vs. mature cells for the final analysis was made using a formal discrimination algorithm (quadratic discriminant analysis functions qda.fit and qda.pred in R) in combination with an expert evaluation.

**Figure 2:**
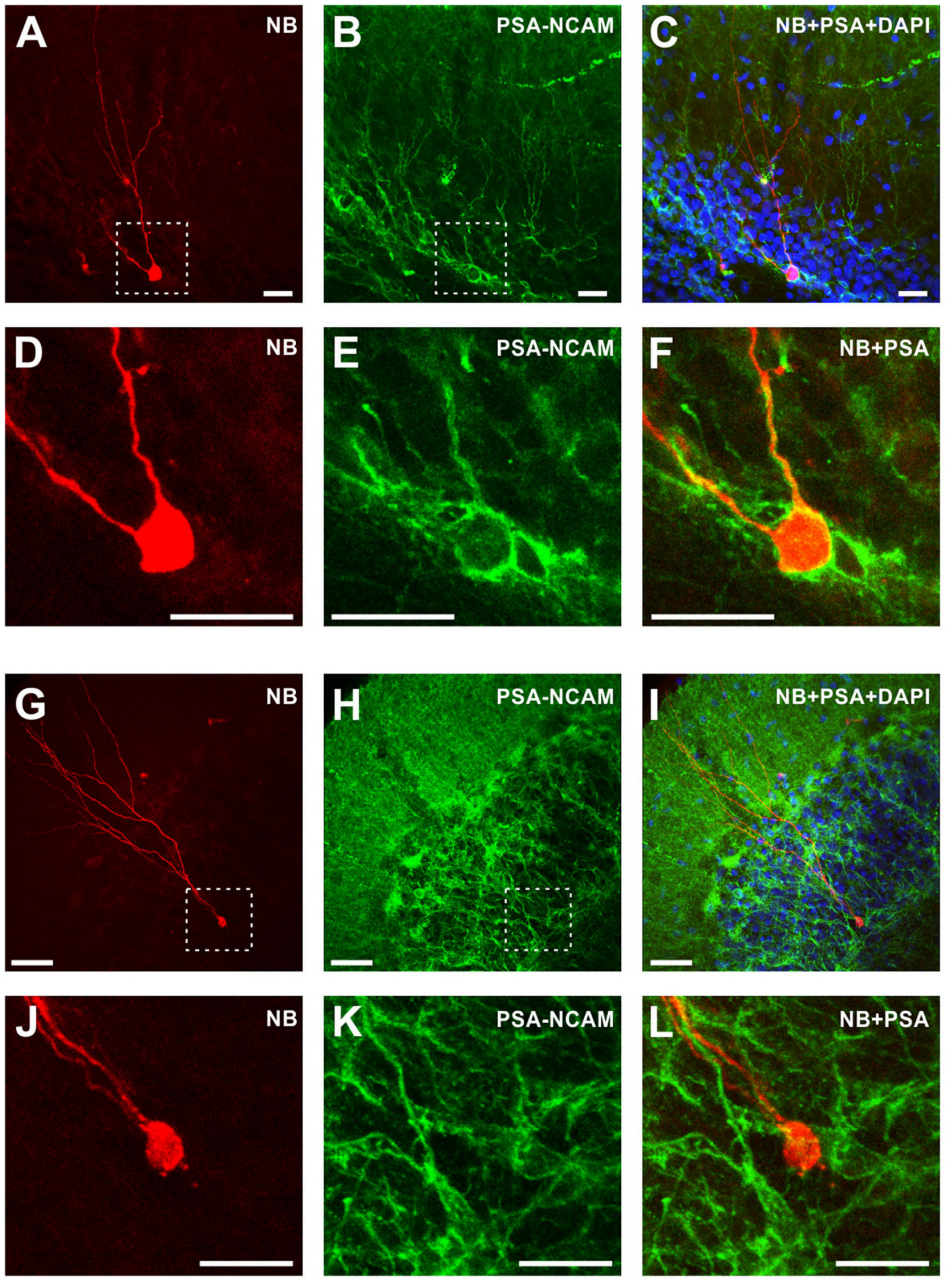
Morphological and immunochemical identification of immature and mature granule cells in the dentate gyrus. Left column **(A,D,G,J)** Fluorescent images of neurobiotin-labeled immature granule cell with rudimentary dendritic tree **(A,D)** and a mature granule cell with a characteristic developed dendritic tree **(G,J)**. **(D)** and **(J)** show zoom-in of the regions indicated by dashed lines in **(A)** and **(G)**, respectively. Note the difference in scale, 25 μm in **(A)** and 50 μm in **(G)**. Middle column **(B,E,H,K)** Immunohistochemical staining with antibodies against PSA-NCAM of the same preparations as in the left column. Right column **(C,F,I,L)** Superimposition of the images from the left column (morphology), middle column (anti-PSA-NCAM immunostaining), and DAPI staining **(C,I)**. Note that immature granule cell is PSA-NCAM positive **(F)**, while mature granule cell is PSA-NCAM negative **(L)**. Scale bars are 25 μm in **(A–F)** and **(J–L)**; and 50 μm in **(G–I)**.

For the formal classification, we first trained the discrimination algorithm using electrophysiological data from cells stained with neurobiotin and processed for morphological analysis and immunolabelling with antibodies against PSA-NCAM, which is a marker for immature cells (Figure 2). Mature granule cells had a developed dendritic tree of a characteristic shape (Figures 2G,J) and were immunonegative to PSA-NCAM (Figures 2I,L). In contrast, immature cells had a rudimentary, undeveloped dendritic tree (Figures 2A,D) and were immunopositive to PSA-NCAM (Figures 2C,F). Distance from the most distal dendrite to the soma, which we used as a quantitative measure of the morphological difference between the developed dendritic tree of mature cells and undeveloped dendritic tree of immature cells, was markedly larger in mature PSA-NCAM negative cells than in immature PSA-NCAM positive cells (394 ± 49 μm *n* = 6 vs. 207 ± 50 μm *n* = 6, *p* < 0.001). Notably, in this subset of morphologically identified cells, immature cells had higher R_in_ (Figure 1C, 1,074 ± 492 vs. 284 ± 82 MOhm in mature cells, *p* = 0.003), and a more depolarized membrane potential (Figure 1D, −60.7 ± 3.9 mV vs. −70.4 ± 9.2 mV in mature cells, *p* = 0.0393). Higher R_in_ and more depolarized potential in immature as compared to mature granule cells has been consistently reported in prior studies (Schmidt-Hieber et al., 2004; Markwardt et al., 2009; Mongiat et al., 2009; Trinchero et al., 2019b). We used this clear difference between identified immature and mature cells in R_in_ and membrane potential for formal classification of the recorded neurons. After training classification algorithm on data from morphologically identified immature (*n* = 6) and mature (*n* = 6) cells, we used the trained algorithm to compute classes in the whole population of cells (*n* = 86 cells, including 12 morphologically identified and 74 identified with electrophysiological data only).

To verify the assignment to the groups of immature or mature cells for the final analysis, results of the formal classification were complemented by expert evaluation based on the combination of features. We used pairwise scatter plots of electrophysiological parameters such as cell capacitance, membrane time constant, R_in_, membrane potential, PPR, and rising slope of synaptic responses (e.g., Figures 1D–H), and excluded from the final analysis those cells, which were represented by data points located in a “wrong” cloud of points. By combination of features, two cells out of 22 formally classified as immature fell into the point cloud for mature cells; one cell out of 52 cells formally classified as mature fell into the point cloud for immature cells. These 3 cells (out of 74 cells subject to classification by electrophysiological parameters) were excluded from the final analysis.

**Figure 3:**
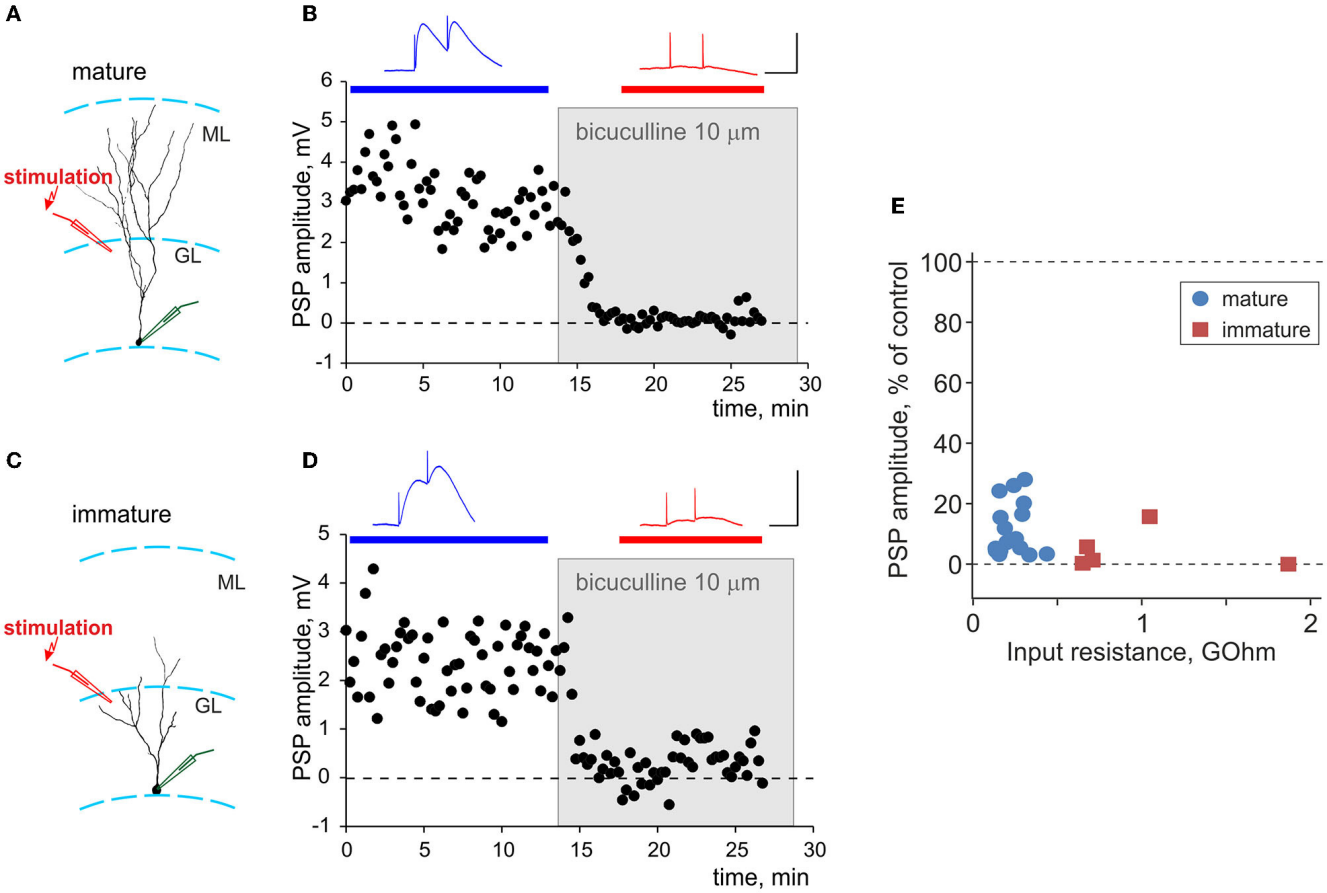
Synaptic responses evoked by local stimulation in the granular layer are GABA-ergic in both mature and immature neurons. **(A,C)** Schemes of the location of a neurobiotin-labeled mature **(A)** and immature **(C)** neurons with the somata in the inner portion of the granule layer (GL), and stimulation electrodes in the outer portion of the granule layer, close to the molecular layer (ML). **(B,D)** Suppression of synaptic responses in mature **(B)** and immature **(D)** neurons by bicuculline. Each time course shows individual PSP amplitudes, with application of 10 μM bicuculline indicated by a gray rectangle. PSPs above the plots are averages over the periods indicated by the horizontal bars of respective color, before and after bicuculline application. Scale bars: 50 ms, 3 mV. **(E)** Averaged amplitude of PSP responses after application of bicuculline, in percentage of control, plotted against input resistance for *n* = 16 mature (blue circle symbols) and *n* = 5 immature (dark red square symbols) neurons.

Compared to mature neurons, immature cells had higher input resistance (1,032 ± 496 MOhm *n* = 26 immature vs. 236 ± 83 MOhm *n* = 57 mature, *p* < 0.001), more depolarized membrane potential (−61.1 ± 4.6 mV immature vs. −74.9 ± 6.6 mV mature, *p* < 0.001), longer membrane time constant (33.3 ± 14.7 ms immature vs. 12.9 ± 4.8 ms mature, *p* < 0.001), and lower capacitance (34.6 ± 10.1 pF immature vs. 56.9 ± 17.0 pF mature, *p* < 0.001). Synaptic responses, evoked by paired-pulses (50 ms inter-pulse interval) applied through a glass pipette placed in the outer portion of granule cell layer (see Figure 3 for placement of stimulation electrode), were also different in immature and mature neurons (Figure 1B). Synaptic responses in immature cells had slower onset dynamics, with PSP slope measured at 10–90% of the amplitude 0.31 ± 0.22 mV/ms in immature (*n* = 20) vs. 3.1 ± 3.3 mV/ms in *n* = 51 mature, *p* < 0.001) and a lower PPR (0.44.6 ± 0.13 immature vs. 0.73 ± 0.27 mature, *p* < 0.001). These PPR values, and lower PPR in immature neurons are consistent with a prior study of maturation of GABA-ergic transmission in new granule cells (Markwardt et al., 2009). PPR is considered to reflect mostly presynaptic mechanisms (Markram et al., 1998; Reyes et al., 1998; Hefft et al., 2002; Zucker and Regehr, 2002; Markwardt et al., 2009; Blackman et al., 2013). Strong paired-pulse depression in immature cells might indicate that mechanisms of synaptic vesicle replenishment are not fully developed at these synapses, but could be also due to immature mechanisms of transmitter clearance, and a stronger influence of presynaptic GABA_B_ receptors on release at new synapses on immature neurons (Markwardt et al., 2009).

The observed differences in electrophysiology of immature and mature cells, verified by morphological and immunochemical data, are consistent with the results of prior studies in rats (Schmidt-Hieber et al., 2004) and mice (Markwardt et al., 2009; Mongiat et al., 2009; Trinchero et al., 2019a). Together, these results show that, using conservative classification criteria, we achieved a robust and reliable segregation of mature and immature cells into two groups.

### Synaptic Responses in Mature and Immature Neurons to Local Stimulation Are Predominantly GABA-Ergic

To evoke synaptic responses, we used local stimulation with an electrode placed in the outer portion of the granule cell layer, above the recording site (Figures 3A,C). Prior research showed that such stimulation evokes GABA-ergic responses in both mature and immature granule cells neurons (Markwardt et al., 2009). To verify GABA-ergic nature of recorded PSP in our experiments, we used a selective GABA_A_-receptor antagonist bicuculline. Bath application of 10 μM bicuculline almost completely suppressed PSP responses in experiments shown in Figure 3B for mature and in Figure 3D for immature neurons. Strong, oftentimes complete suppression of PSPs by bicuculline was consistently observed in all experiments, as illustrated in Figure 3E in which the amplitude of PSP response after application of bicuculline is plotted against R_in_ for *n* = 16 mature and *n* = 5 immature neurons. On average, 10 μM bicuculline suppressed PSP responses in mature cells to 12 ± 9% of control and in immature cells to 5 ± 7 % of control. There was no significant difference between suppression in mature vs. immature cells (*p* = 0.088).

Thus, consistent with prior work (Markwardt et al., 2009), synaptic responses evoked in both mature and immature granule cells by local stimulation in the outer portion of the granule cell layer were largely GABA_A_-ergic (on average, GABA_A_ component contributed 95% in immature and 88% in mature neurons), with negligible or little contribution of other mediators. For this reason, and because recent evidence shows involvement of glutamatergic mechanisms in regulation of synaptic plasticity at GABA-ergic synapses (e.g., Chiu et al., 2018), we did not block excitatory transmission in the main series of plasticity experiments (refer to the following sections for a control series of experiments in the presence of AMPA blocker CNQX).

### Bursts of Action Potentials Induce Distinct Heterosynaptic Plasticity in Mature and Immature Neurons

**Figure 4:**
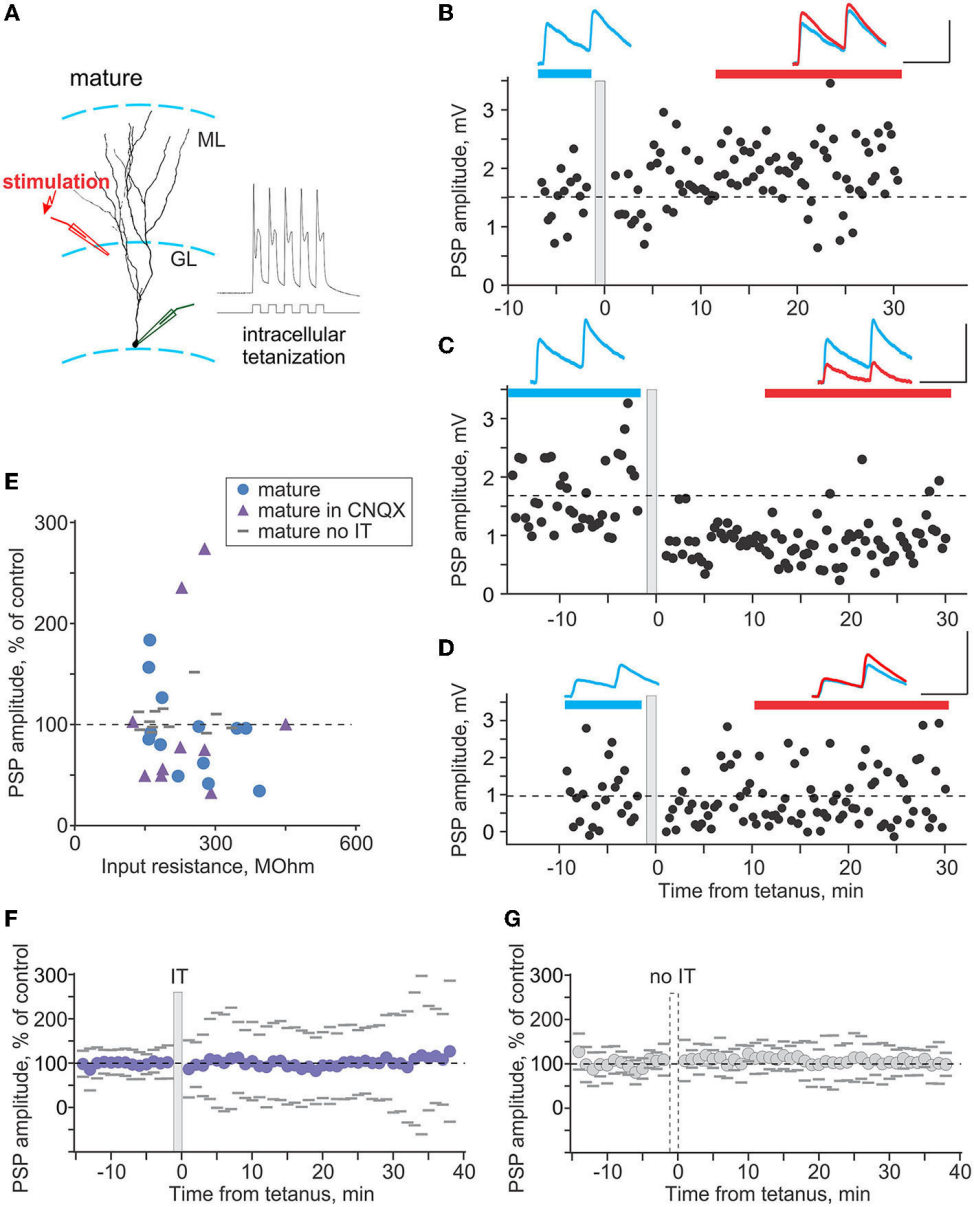
Plasticity induced by intracellular tetanization in mature neurons. **(A)** A scheme of recording from a mature neuron and location of stimulation electrode, and response of the neuron to one burst of intracellular tetanization (5 brief depolarizing pulses at 100 Hz). **(B–D)** Three example experiments in which intracellular tetanization induced LTP **(B)**, LTD **(C)**, or did not lead to changes of synaptic responses in mature neurons **(D)**. Each time course shows individual PSP amplitudes; dashed horizontal line shows mean PSP amplitude before intracellular tetanization (gray vertical bar). PSPs above the plots are averages over the periods indicated by the horizontal bars of respective color, before and after intracellular tetanization. Scale bars: 50 ms, 2 mV. **(E)** PSP amplitude in percentage of control after intracellular tetanization (or no tetanization), plotted against input resistance. Intracellular tetanization was applied in experiments without synaptic blockers (*n* = 13 blue circle symbols), with 10 μm CNQX in the recording medium (*n* = 10, violet triangle symbols). In *n* = 12 experiments without synaptic blockers, no tetanization was applied (gray horizontal dash symbols). **(F,G)** Summary time course of PSP amplitude changes, in percentage of control, in experiments with intracellular tetanization [**(F)**, *n* = 23 cells, “IT”] and without intracellular tetanization [**(G)**, *n* = 12 cells, “no IT”]. For each cell, PSP amplitudes were normalized to control and then average responses over 1 min periods were calculated. These 1-min averages were used to calculate average (circle symbols) and SD across cells. Gray dashes show ±SD.

**Figure 5:**
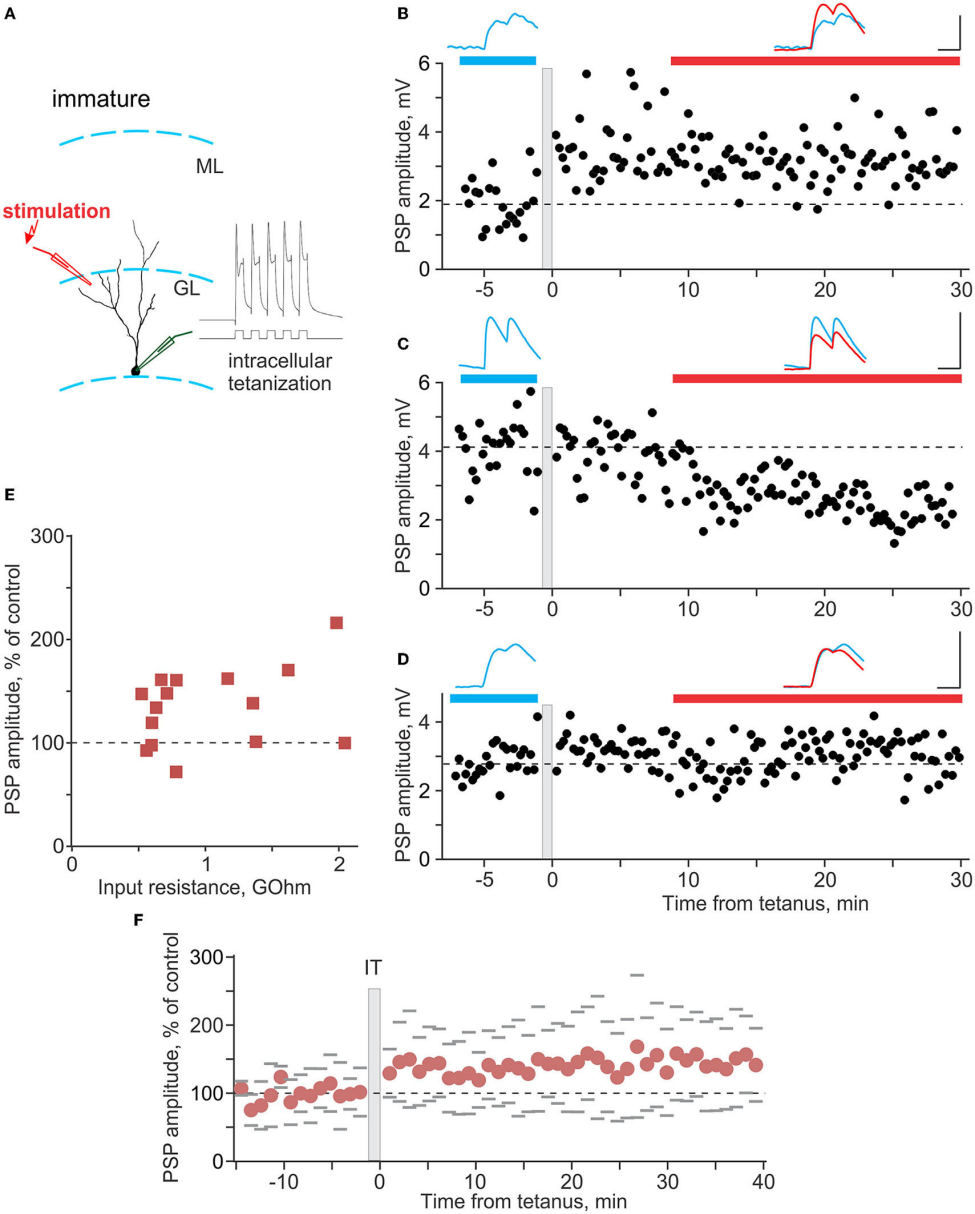
Plasticity induced by intracellular tetanization in immature neurons. **(A)** A scheme of recording from an immature neuron and location of stimulation electrode, and response of the neuron to one burst of intracellular tetanization (5 brief depolarizing pulses at 100 Hz). **(B–D)** Three example experiments in which intracellular tetanization induced LTP **(B)**, LTD **(C)**, or did not lead to changes of synaptic responses in immature neurons **(D)**. Each time course shows individual PSP amplitudes, and dashed horizontal line shows mean PSP amplitude before intracellular tetanization (gray vertical bar). PSPs above the plots are averages over the periods indicated by the horizontal bars of respective color, before and after intracellular tetanization. Scale bars: 50 ms; 2 mV in **(B)**, 4 mV in **(C,D)**. **(E)** PSP amplitude after intracellular tetanization, in percentage of control, plotted against input resistance for *n* = 15 immature neurons. **(F)** Summary time course of PSP amplitude changes, in percentage of control, in experiments with intracellular tetanization for *n* = 15 immature neurons. For each cell, PSP amplitudes were normalized to control and then average responses over 1 min periods were calculated. These 1-min averages were used to calculate average (circle symbols) and SD across cells. Gray dashes show ±SD.

To study heterosynaptic plasticity of synaptic transmission to mature and immature granule cells, we used an established protocol of intracellular tetanization (Volgushev et al., 1994, 1997, 2000; Chistiakova et al., 2015). Intracellular tetanization consisted of bursts of postsynaptic action potentials evoked by depolarizing pulses applied to the cell through the recording electrode, without presynaptic stimulation. Each burst consisted of five depolarizing pulses of 5 ms duration applied at 100 Hz, with the current amplitude (0.1–1.5 nA) adjusted so that each pulse evoked an action potential (Figures 4A, 5A). Three trains (one per minute) of 10 bursts (at 1 Hz) of depolarizing pulses were applied. Prior research showed that intracellular tetanization can induce bidirectional plasticity (LTP or LTD) in excitatory and inhibitory neurons in the visual cortex (Volgushev et al., 2000, 2016; Bannon et al., 2017; Chistiakova et al., 2019; Chasse et al., 2021), in the auditory cortex (Lee et al., 2012), and in the hippocampus (Kuhnt et al., 1994). Similar protocols, consisting of postsynaptic spikes without presynaptic activation, can induce plasticity of inhibitory transmission in visual (Kurotani et al., 2003, 2008) and somatosensory (Lourenço et al., 2014) cortex. Because such protocols could induce bidirectional plastic changes, they allow to study the balance of potentiation and depression, and to compare the LTP/LTD balance between experimental groups.

In mature granule cells (Figure 4A), intracellular tetanization induced bidirectional plasticity. Figure 4 shows example experiments in which PSP responses expressed LTP (Figure 4B), LTD (Figure 4C), or did not change (Figure 4D) after intracellular tetanization. In each panel (Figures 4B–D), time course shows amplitudes of individual responses. Timing of the intracellular tetanization is indicated by the gray vertical bar. PSP responses averaged over the periods before (blue) and after (red) tetanization are shown in respective colors, and are superimposed for comparison. In the first series of experiments with no synaptic blockers (*n* = 13), LTP was observed in 3 cases, LTD in 6 cases, and in the remaining 4 experiments, PSP amplitude did not change (Figure 4E, blue circle symbols). While synaptic responses in our recording conditions are mostly mediated by GABA_A_ receptors, as shown by the results of bicuculline application, we nevertheless performed another series of experiments with 10 μm CNQX in the recording medium, to ensure that AMPA-mediated components do not contribute to the observed effects. In these experiments (*n* = 10), LTP was observed in 2 cases, LTD in 6 cases, and in the remaining 2 experiments, PSP amplitude did not change (Figure 4E, violet triangle symbols). Because there was no difference between the effects of intracellular tetanization in experimental series with and without CNQX, neither in the overall changes of PSP amplitude (93 ± 43% of control vs. 105 ± 82% of control, *p* = 0.66) nor in the frequency of occurrence of LTP, LTD and no change (3-6-4 vs. 2-6-2, chi-square test *p* = 0.51), results from these two series were pooled together. Note that because of stability criteria for including cells in the final analysis, observed LTP and LTD of synaptic responses cannot be attributed to the shifts of the membrane potential or changes of input resistance.

In additional series of experiments, we tested whether observed changes of synaptic transmission were indeed caused by intracellular tetanization. We recorded PSPs on the same schedule in 12 experiments, but did not apply intracellular tetanization. No significant changes were observed in 11 of these experiments, and response amplitude increased in 1 case (Figure 4E, gray dash symbols). Frequency of occurrence of PSP changes was clearly different in experiments with vs. without the tetanization (chi-square test *p* < 0.001). Intracellular tetanization induced balanced changes, with no net change of PSP amplitude averaged over all experiments (Figure 4F). However, variance of the post-tetanization amplitude changes across cells is expected to be high because the occurrence of LTP in some and LTD in other neurons introduces additional variability to spontaneous changes (see Chistiakova et al., 2014, 2015). In contrast, in experiments without tetanization (Figure 4G), although there is also no net change of PSP amplitude, the across-cells variance of response changes is expected to remain low, because only spontaneous (random) changes of response amplitudes occur. Indeed, variance of PSP amplitude changes after intracellular tetanization was significantly higher than in experiments without tetanization (62% *n* = 23 vs. 17% *n* = 12, F-test *p* < 0.001; compare Figures 4F,G), confirming that plastic changes after intracellular tetanization could not be explained by spontaneous fluctuations of synaptic responses.

Together, these results show that in mature neurons, intracellular tetanization induced bidirectional (both LTP and LTD) balanced plasticity, which was not dependent on the function of AMPA-receptors.

In immature granule cells (Figure 5A), intracellular tetanization also induced bidirectional changes, with LTP (Figure 5B), LTD (Figure 5C), or no changes of PSP amplitudes (Figure 5D) observed in individual experiments. However, the overall picture of plasticity was very different from mature neurons. LTP was observed in 10 out of 15 experiments (to 156 ± 26% of control), LTD in only 1 case, and PSP amplitude did not change in 4 experiments (Figure 5E). As a consequence of the high frequency of occurrence of LTP, gross average across all experiments revealed a significant potentiation to 135 ± 38% of control (Figure 5F, *n* = 15, *p* = 0.008).

**Figure 6:**
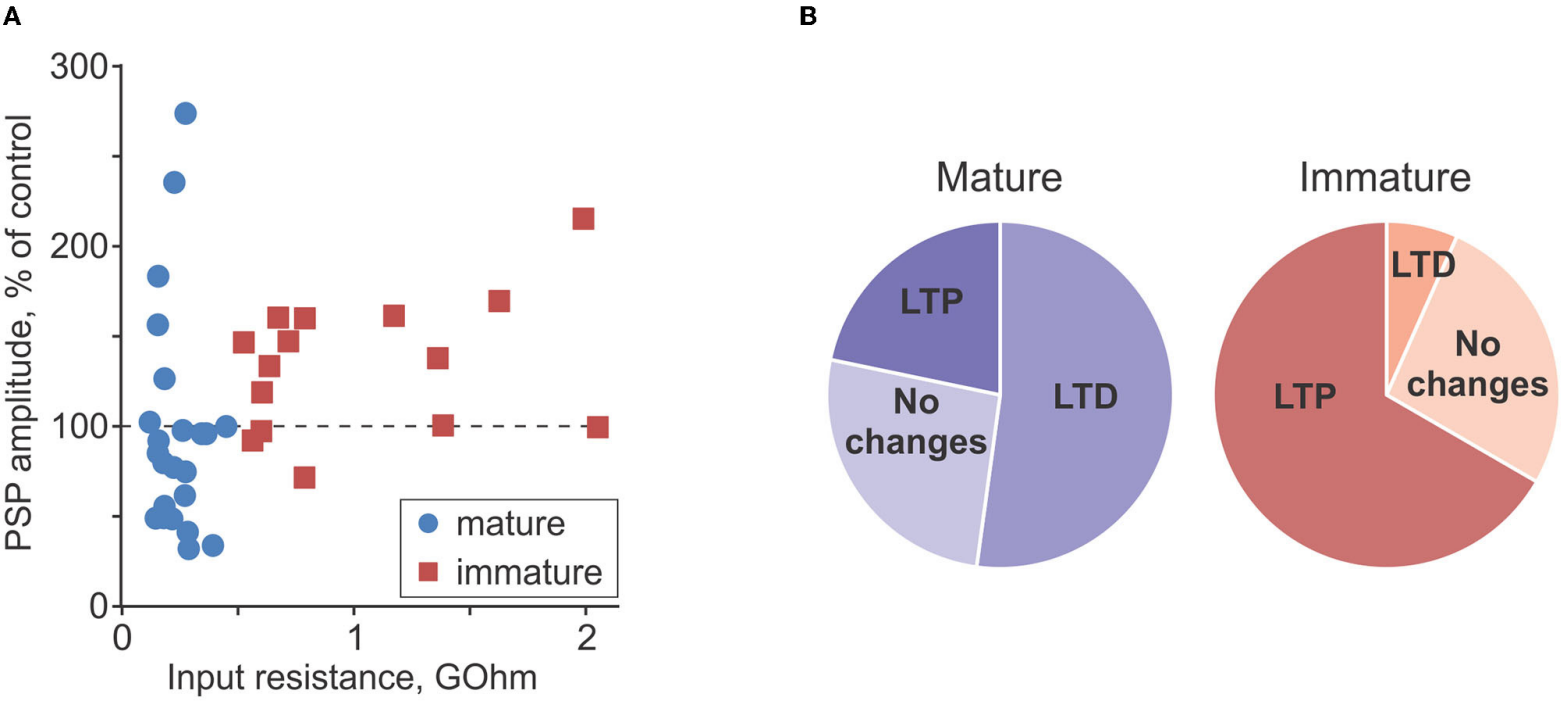
Comparison of plasticity in mature and immature neurons. **(A)** PSP amplitude after intracellular tetanization, in percentage of control, plotted against input resistance for *n* = 23 mature (blue circle symbols) and *n* = 15 immature (dark red square symbols) neurons. Same data as in Figures 4E, 5E. **(B)** Pie plots showing frequency of occurrence of LTP, LTD, and no changes after intracellular tetanization in mature (*n* = 23, left, blueish colors) and immature (*n* = 15, right, reddish colors) neurons.

Figure 6 summarizes the difference in plasticity between mature and immature neurons. In Figure 6A, in which PSP amplitude after intracellular tetanization is plotted against R_in_, data points representing mature neurons (blue circles) are distributed both above (LTP) and below (LTD) the 100% of control amplitude (horizontal dashed line). In contrast, data points representing immature neurons (red squares) are located mostly above the 100% of control amplitude line, showing predominance of LTP. Notably, for the pooled data from plasticity experiments, there was a tendency for a positive correlation between R_in_ and PSP amplitude change (*r* = 0.31, *p* = 0.054, *n* = 38; all cells, mature and immature). Pie plots in Figure 6B illustrate higher frequency of LTP in immature as compared with mature neurons (*p* < 0.001, chi-square test). Thus, overall potentiation and predominance of LTP in immature neurons was due to the higher frequency of occurrence of LTP, while the amplitude of LTP in individual inputs was similar in mature and immature neurons (156 ± 26% of control vs. 196 ± 60% of control, *p* = 0.21).

**Figure 7:**
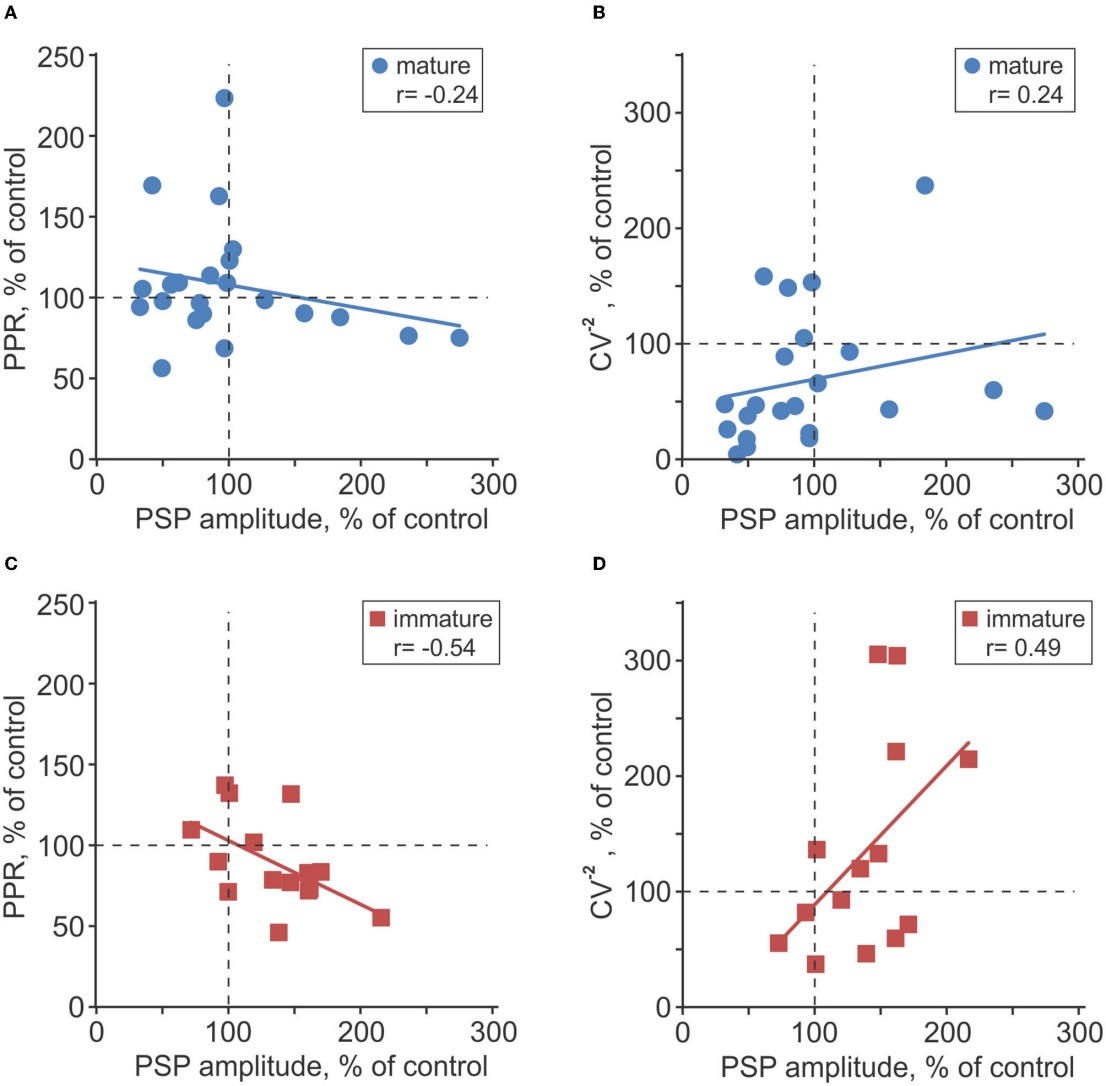
Changes of release probability indices associated with plasticity in mature and immature neurons. **(A–D)** Changes of the paired-pulse ratio [PPR; **(A,C)**] and inversed coefficient of variation [CV^−2^; **(B,D)**] plotted against PSP amplitude changes after intracellular tetanization in *n* = 23 mature (A,B) and *n* = 15 immature **(C,D)** neurons. All changes are calculated in percentage of respective values in control, before intracellular tetanization.

To assess possible involvement of presynaptic mechanisms in observed plastic changes, we measured two indices of presynaptic release, PPR and the inverse of the coefficient of variation (CV^−2^), and their changes after plasticity induction (Voronin, 1993; Markram et al., 1998; Reyes et al., 1998; Zucker and Regehr, 2002; Blackman et al., 2013). In immature neurons, changes of PPR were inversely correlated with changes of PSP amplitude after intracellular tetanization (*r* = −0.54, *p* = 0.039, *n* = 15; Figure 7C), and changes of CV^−2^ had a tendency to correlate positively with PSP amplitude changes (*r* = 0.49, *p* = 0.062, *n* = 15; Figure 7D). These correlations indicate involvement of presynaptic mechanisms in heterosynaptic plasticity in immature neurons. In mature neurons, correlations did not reach significance level (*r* = −0.24, *p* > 0.1 for PPR changes, and *r* = 0.24, *p* > 0.1 for CV^−2^ changes, *n* = 23; Figures 7A,B).

Prior work on heterosynaptic plasticity of excitatory inputs to neurons in visual and auditory cortex found that the direction and magnitude of amplitude changes were significantly correlated with initial PPR (Volgushev et al., 2000, 2016; Lee et al., 2012; Bannon et al., 2017; Chistiakova et al., 2019). In the present study of plasticity of inhibitory transmission to granule cells, no such correlation was found, neither for mature (*r* = 0.10; *p* > 0.1), nor for immature neurons (*r* = 0.09; *p* > 0.1).

## Discussion

Results of the present study show (1) that GABA-ergic inputs to both immature and mature granule cells in the dentate gyrus are plastic, (2) that heterosynaptic, non-associative plasticity at these inputs can be induced by postsynaptic spiking without presynaptic activation, and (3) reveal a shift of the properties of non-associative plasticity with maturation of newly generated granule cells: while both immature and mature neurons could express either LTP or LTD at individual inputs, there was a clear predominance of LTP in immature cells but about balanced changes in mature neurons.

These findings add several important pieces to the picture of plasticity in new-born neurons in adult dentate gyrus. Prior work demonstrated that associative plasticity of excitatory inputs is enhanced in newly generated immature granule cells as compared to mature neurons. In immature neurons, threshold for induction of plasticity by theta-burst stimulation of excitatory inputs is lower (Schmidt-Hieber et al., 2004; Ge et al., 2007b), and in 1 to 1.5-month-old neurons, the magnitude of LTP is higher than in mature cells (Ge et al., 2007b). Here, we show that GABA-ergic inputs to immature and mature granule cells can undergo non-associative bidirectional plasticity, and that features of non-associative plasticity at GABA-ergic synapses change with maturation of granule cells. However, these changes are quite different from changes of plasticity at excitatory synapses. While the percentage of GABA-ergic inputs that expressed plasticity was not different between immature (LTP or LTD in 73% of experiments, 11/15 cells) and mature cells (LTP or LTD in 74% of experiments, 17/23 cells), the balance between potentiation and depression changed dramatically. In immature neurons, LTP strongly dominated, resulting in net potentiation after an episode of strong purely postsynaptic activity. In mature neurons, such non-associative changes were balanced. For pooled data from immature and mature cells, there was a tendency for a positive correlation between R_in_ and PSP amplitude change. Because R_in_ is an established correlate of the maturation of newly generated granule cells, this correlation indicates that a shift from the predominance of potentiation in immature cells to balanced non-associative plastic changes in mature neurons reflects an intrinsic property of the cell maturation process. This also suggests that the shift might occur gradually during the maturation of new cells and their integration in the existing circuitry. Of note is also that net potentiation in immature neurons was due to a more frequent occurrence of LTP, while the magnitude of LTP in potentiated inputs was not different in immature and mature cells. This indicates that underlying mechanism might be a developmental change of the triggering or/and thresholds for induction of LTP and LTD, such as a shift in the balance between R-type calcium channels that mediate potentiation and L-type channels that mediate depression of GABA-ergic transmission in L5 pyramidal cells (Kurotani et al., 2008), or expression of other receptors regulating LTP/LTD balance of heterosynaptic changes, such as adenosine A1 receptors at excitatory synapses onto cortical neurons (Bannon et al., 2017; Chasse et al., 2021).

Calcium rise is proposed as a trigger of associative plasticity at excitatory synapses to mature and immature granule cells in the dentate gyrus (Schmidt-Hieber et al., 2004). An increase of intracellular calcium concentration might also be a trigger for non-associative plasticity at GABA-ergic synapses, observed in the present study. The role of the rise of intracellular calcium concentration produced by postsynaptic activity without presynaptic activation in triggering heterosynaptic plasticity in granule cells is consistent with results of prior studies of heterosynaptic plasticity at excitatory inputs to pyramidal neurons and inhibitory cells in visual and auditory cortex (Volgushev et al., 2000; Balaban et al., 2004; Lee et al., 2012; Chistiakova et al., 2019) and in inhibitory inputs to L5 pyramids in visual and somatosensory cortex (Kurotani et al., 2003, 2008; Lourenço et al., 2014). Indeed, intracellular tetanization produced an increase of intracellular calcium concentration in layer 2/3 pyramidal neurons (Balaban et al., 2004). A decrease of the calcium rise by a blockade of NMDA receptors (Balaban et al., 2004) shifted the balance of heterosynaptic plasticity toward potentiation (Chistiakova et al., 1999), and partial buffering of intracellular calcium with EGTA hindered the induction of heterosynaptic plasticity, resulting in both lower frequency of occurrence and lower magnitude of LTP and LTD (Lee et al., 2012). The role of specific sources of calcium influx and rise, and specific intracellular cascades mediating heterosynaptic plasticity of GABA-ergic transmission in granule cells, as well as changes of these mechanisms underlying the observed shift of the properties of heterosynaptic plasticity with maturation remain open questions for further research.

Non-associative plasticity of GABA-ergic inputs to immature granule cells was accompanied by changes of presynaptic release indices (PPR and CV^−2^), indicating an involvement of presynaptic mechanisms in plasticity expression. Because spikes during intracellular tetanization were induced in only one cell, and unitary connections from granule cells to interneurons are typically too weak to induce postsynaptic spikes (Scharfman et al., 1990; Geiger et al., 1997; Espinoza et al., 2018), plasticity induction protocol was purely postsynaptic. Presynaptic changes after a purely postsynaptic induction protocol imply retrograde signaling. Candidate mechanisms for retrograde signaling suggested by prior studies of heterosynaptic plasticity include signaling employing NO (Volgushev et al., 2000; Kurotani et al., 2008; Lee et al., 2012; Lourenço et al., 2014), BDNF (Inagaki et al., 2008; Kuczewski et al., 2008), and astrocyte-released ATP (Chen et al., 2013b). Whether these and/or other retrograde signaling pathways are mediating presynaptic changes of GABA-ergic transmission in immature granule cells remains to be clarified.

An important question is at what age of newly generated neurons the shift of non-associative plasticity from predominantly potentiation to balanced changes occurs, and thus what was the age of the immature neurons in our experiments. Presence of dendrites reaching from the inner portion of granule cell layer (recording site, soma location) to the molecular layer, and clear GABA-ergic PSP responses evoked by electric stimulation indicate that immature neurons in our experiments were >14 days old, and values of R_in_ suggest an age range of 19–24 days (Ge et al., 2006; Mongiat et al., 2009; Dieni et al., 2013; Trinchero et al., 2019a). This age range is also compatible with the results of immunostaining against PSA-NCAM, with immunopositive immature, and immunonegative mature neurons. Indeed, while many studies report that PSA-NCAM expression in immature neurons terminates by the end of the second week, there is also evidence that 3 or 4-week-old cells in dentate gyrus of rats can be immunopositive for PSA-NCAM (Seki, 2002). These considerations suggest that the bias of non-associative plasticity of GABA-ergic transmission toward potentiation is still present during the third and fourth weeks of the maturation of newborn granule cells, and the shift to balanced, mature-type heterosynaptic plasticity takes place after the fourth week.

### Open Questions and Limitations of the Present Study

Results of the present study open up a whole range of new questions, which are important for understanding mechanisms of integration of newborn granule cells into existing circuitry, but go beyond the scope of the present study.

At the first place, there are questions concerning specific mechanisms of heterosynaptic plasticity at GABA-ergic synapses onto immature and mature granule cells. Is this form of plasticity triggered by the rise in intracellular calcium concentration, as it is the case in most of other cells and synapses? What are sources of the calcium rise, and what levels of intracellular calcium are necessary for triggering plasticity? Could strong but subthreshold depolarization be sufficient to reach these levels, or generation of action potentials is needed for recruitment of essential calcium sources? Finally, what mechanisms mediate the shift in the properties of heterosynaptic plasticity at GABA-ergic synapses during maturation of adult-born new granule cells? Is there a change of the thresholds for LTD/LTP induction, a change of the sources of calcium rise, or changes of intracellular cascades that are triggered by calcium?

Another group of open questions concerns the interaction between non-associative and associative plasticity at GABA-ergic and glutamate-ergic synapses. This study revealed non-associative plasticity, and enhanced non-associative potentiation at GABA-ergic synapses in newborn granule cells. Prior work described enhanced associative plasticity of glutamate-ergic synapses to immature granule cells. It remains to be elucidated whether there is associative plasticity at GABA-ergic, and non-associative plasticity at glutamate-ergic synapses to immature granule cells, what are the mechanisms and rules for their induction, and how these diverse forms of plasticity interact during maturation of newborn granule cells.

Resolving these questions would be essential for understanding the role of diverse forms of plasticity and their interaction in a broader context of survival and integration of newborn granule cells in the existing circuitry, and ultimately their contribution to maintenance and refinement of hippocampal function.

### Outlook: A Role for Non-Associative Heterosynaptic Plasticity

Prior experimental and computational analysis of heterosynaptic plasticity at excitatory synapses postulated its role in maintaining the balance of synaptic changes, preventing runaway dynamics of synaptic weights and activity, and enhancing synaptic competition (Chen et al., 2013a; see Chistiakova et al., 2015 and Bannon et al., 2020 for review; Volgushev et al., 2016; Bannon et al., 2017). Computational studies predicted the role of heterosynaptic plasticity in learning on sequential tasks. Evidence in support of this prediction for organism-level learning was provided in a recent work demonstrating that impairment of heterosynaptic plasticity in adenosine A1 receptor knockout mice is accompanied by a selective impairment of re-learning on consequent tasks but no impairment in the initial learning (Chasse et al., 2021). Results of the present study propose an additional role, which non-associative heterosynaptic plasticity could play at GABA-ergic synapses during maturation of newborn granule cells in the adult dentate gyrus. With potentiation-biased non-associative plasticity, activation and firing of immature granule cells could promote strengthening of existing GABA-ergic synapses and, *via* retrograde signaling mechanisms, formation of new synapses. Such mechanism might play an important role in reconciling the requirement for cell activity, which is needed for survival of newborn neurons and promoting formation of excitatory synapses (Tashiro et al., 2006; Ge et al., 2007a; Dieni et al., 2013), with the requirement to build a strong GABA-ergic input to surviving cells, which is necessary to achieve a robust GABA-ergic inhibition, tight inhibitory control, and sparse spiking activity of mature granule cells (Dieni et al., 2013).

## Data Availability Statement

The raw data supporting the conclusions of this article will be made available by the authors, without undue reservation.

## Ethics Statement

The study and all procedures involving animals were reviewed and approved by the Ethical Committee of the Institute of Higher Nervous Activity and Neurophysiology, Russian Academy of Sciences.

## Author Contributions

MV and AM designed experiments. NS and AM carried out experiments. NS, MV, and AM processed, analyzed the data, and wrote the manuscript. All authors significantly contributed to the article and approved the submitted version.

## Funding

This study was supported by the Russian Science Foundation (grant #20-15-00398 to AM).

## Conflict of Interest

The authors declare that the research was conducted in the absence of any commercial or financial relationships that could be construed as a potential conflict of interest.

## Publisher's Note

All claims expressed in this article are solely those of the authors and do not necessarily represent those of their affiliated organizations, or those of the publisher, the editors and the reviewers. Any product that may be evaluated in this article, or claim that may be made by its manufacturer, is not guaranteed or endorsed by the publisher.
